# Dairy intensification: Drivers, impacts and alternatives

**DOI:** 10.1007/s13280-019-01177-y

**Published:** 2019-05-04

**Authors:** Nathan Clay, Tara Garnett, Jamie Lorimer

**Affiliations:** 1 School of Geography and the Environment, University of Oxford, South Parks Road, Oxford, OX1 3QY UK; 2 Food Climate Research Network, Environmental Change Institute, University of Oxford, South Parks Road, Oxford, OX1 3QY UK

**Keywords:** Agricultural intensification, Agroecology, Food system, Multifunctional agriculture, Organic, Sustainable intensification

## Abstract

Dairy production systems have rapidly intensified over the past several decades. Dairy farms in many world regions are larger and concentrated in fewer hands. Higher productivity can increase overall economic gains but also incurs site-specific social and environmental costs. In this paper, we review the drivers and impacts of dairy intensification. We identify in the literature four prominent concerns about dairy intensification: the environment, animal welfare, socioeconomic well-being, and human health. We then critically assess three frameworks—sustainable intensification, multifunctionality, and agroecology—which promise win–win solutions to these concerns. We call for research and policy approaches that can better account for synergies and trade-offs among the multiple dimensions of dairy impacts. Specifically, we suggest the need to (1) consider dairy system transitions within broader processes of social-environmental change and (2) investigate how certain framings and metrics may lead to uneven social-environmental outcomes. Such work can help visualize transformations towards more equitable, ethical, and sustainable food systems.

## Introduction

Dairy farming has rapidly intensified over the past 50 years (FAO 2018a). Trends towards fewer larger farms have accelerated further in the past few decades as dairy producers struggle to compete in the burgeoning global marketplace for milk and dairy products (Freidberg 2009). Dairy intensification has complex and uneven effects on human–environment systems. Growing recognition of the negative impacts on the environment, animal welfare, equitable rural development, and human health has led to efforts to envision more sustainable and just dairy systems (Jay 2006). This paper speaks to an emerging literature on dairy intensification, considering drivers, impacts, and alternatives. It focuses on major temperate dairy producing regions of the European Union (EU), North America (NA), and Australia and New Zealand (ANZ).

Since the Second World War, dairy intensification—increased milk output relative to inputs of feed, labour, land, or herd size—has been the dominant trajectory of dairy system change (Jay and Morad 2007). This *productivist* mindset—where increasing efficiency to enhance revenue from agricultural products is the primary objective—continues to inform dairy system policies in the EU, NA, and ANZ (Blayney 2002; McGregor and Houston 2017). If trends continue, global production of dairy products is expected to rise by 22% over the next decade, with dairy intensification likely to accelerate worldwide (FAO 2018a). Moreover, in many parts of the EU, NA, and ANZ, costs of milk production have risen while market price has dropped due to overproduction and retailer control of supply chains (MacDonald et al. 2016). A result of this is that thousands of farms go out of business every year (USDA 2018).

Current modes of dairy intensification are widely recognized to generate negative impacts along multiple dimensions: the environment (Del Prado et al. 2013), animal welfare (Koeck et al. 2014), human health (Westhoek et al. 2014), and rural livelihoods and well-being (Flaten 2002). In reviewing the literature, we find that research on the effects of dairy system intensification tends to consider just one or two of these dimensions. This can obscure understanding of the combined effects across these dimensions. Existing assessments of the impacts of dairy intensification tend to sit within disciplinary silos. In addition, this research tends to present static assessments of dairy systems rather than unpacking processes of human–environment change over time. This prevalence of mono-dimensional studies could wrongly indicate to policymakers that targeting a single set of goals can be enough to address the complex issues of dairy intensification. This in turn risks reinforcing an already single-issue approach to decision making in food system governance.

We suggest that it is essential to consider how the effects of dairy intensification can occur synergistically (e.g. livelihoods and environment can be simultaneously improved or worsened) or as trade-offs (e.g. enhanced economic efficiency may come at the expense of human health). Recent work has considered the political economic and ecological complexities surrounding rapid intensification in the meat industry (Emel and Neo 2015; Neo and Emel 2017). However, we find that similar work on dairy systems has been sparse. To work towards a more comprehensive understanding of dairy system transitions, this paper considers three solutions proposed by different sets of stakeholders for mitigating the negative impacts of intensified production systems: sustainable intensification, multifunctionality, and agroecology.

Sustainable intensification, in brief, denotes an aim of increasing productivity while simultaneously decreasing the negative environmental effects of conventional farming practices (Garnett et al. 2013). Agricultural multifunctionality refers to efforts to derive diverse benefits from agroecosystems that extend beyond production of food and fibre to include environmental services (e.g. carbon sequestration, biodiversity, and water quality) and maintenance of social-cultural processes (e.g. cultural landscapes and family farming units) (Wilson 2007). Agroecology emphasises the context-specific nature of agroecosystems and considers how ecological principles can help achieve goals of sustainability and social equity. We find that these frameworks have been inadequately applied to thinking about dairy system transitions. To initiate a dialogue on their potential value for policy and research, we consider to what degree these different framings help address the multiple human–environment dimensions of dairy intensification and how they may shape alternative trajectories for dairy systems.

This paper first documents the drivers of dairy system intensification and considers the human–environment impacts in four main areas of concern: the environment, animal welfare, human well-being, and human health. It then considers whether the framings of sustainable intensification, multifunctional agriculture, and agroecology are well positioned to address the multidimensional human–environment aspects of dairy systems and what sorts of system transformations these framings offer. We conclude by arguing for the need to (1) consider dairy landscapes within broader processes of socioecological change and (2) investigate how power imbalances can lead to uneven human–environment outcomes amid dairy system transitions.

## Mapping dairy intensification

### Intensification and concentration in dairy farming

Agricultural intensification is rooted in a narrative of agricultural modernization, which defines progress in terms of increasing efficiency and productivity. In the EU, NA, and ANZ, the drive to enhance the economic efficiency of dairy production has guided dairy research and policy over the past 70 years (McGregor and Houston 2017). Policies such as the US Farm Bill and the EU’s Common Agriculture Policy (CAP) have long incentivized dairy farm specialization and encouraged further scientific and technological innovations in agricultural engineering. This policy mandate of intensification centres on increasing the adoption of technologies (e.g. breeding and antibiotics) and use of inputs (e.g. commercially prepared feed) as well as substituting human labour with mechanized feeding and milking (Blayney 2002).

Dairy systems in the EU, NA, and ANZ intensified sharply following the Second World War (Freidberg 2009). While livestock farmers had traditionally produced both meat and dairy, specialized dairy farms became increasingly common. Shifts from pasture-based to confinement feeding systems (first in NA and later in the EU) further revolutionized the dairy industry by enabling consistent production year-round to support growing urban markets for milk (Dupuis 2002). These processes facilitated enormous gains in milk productivity at the level of individual cows, farms, and dairy production regions. As an example, the US dairy herd in 2001 produced three times as much milk as in 1950, even with 30% fewer cows (Blayney 2002). Annual yields per cow now commonly average more than 8000 litres across temperate regions (FAO 2018a).

Dairy system intensification comprises practices at farm-level, at regional levels, and at the level of individual animals. Farm specialization and mechanization strategies emphasize increasing milk production through larger herds, breeding technologies, indoor housing/feeding, energy and protein-dense commercial feeds, antibiotics and growth hormones (in NA), specialized staff or machines. Cows are artificially inseminated at a young age and milked for only a few years until productivity drops due to the steep declines in animal health caused by continuous pregnancy and lactation (Oltenacu and Broom 2010). In very intensive operations, cows are often kept indoors, sometimes year-round, with stall-feeding regimes of imported cereals and oilseed proteins to ensure steady milk production. Thus, intensive dairy operations rely heavily on external inputs: feed that is produced off farm and transported often long distances, water for animals and pasture irrigation, and milking and waste management infrastructure. Dairy production is concentrated regionally in areas with favourable political economic and ecological conditions (Blayney 2002). In major dairy areas, private or state-run companies and cooperatives (e.g. Muller-Wiseman in the UK, Fonterra in NZ) often emerge, putting up capital investment in technologies infrastructure (e.g. breeding, veterinary care, milk processing, and transportation) that further stimulate dairy intensification at a regional level (Jay and Morad 2007).

### Patterns and drivers of dairy intensification

Intensification has occurred together with specialization, meaning fewer dairy farms and larger herd size per farm. In the USA, for example, there were around 640 000 dairy farms in 1970 (Blayney 2002). By 2017 only about 40 000 remained (USDA 2018). In the EU, NA, and ANZ, a clear majority of milk is now produced on relatively few larger farms. While the degree and timing of intensification and concentration vary substantially across and within dairy producing regions, a steep acceleration has occurred over the past two decades. In the USA, where intensification and concentration began relatively early, there was a sharp increase between 1997 (when half of all cows were in herds larger than 140) and 2012, by which time more than half of cows were in herds of more than 900 (MacDonald et al. 2016). Intensification has occurred more recently in the EU, with notable acceleration over the past 20 years. In the UK, for example, between 1995 and 2017 the number of dairy farms fell from around 35 000 to 13 000 while average herd size tripled (Dairy UK 2017). Intensification has been less extreme in NZ, where from 1990 to 2012 the number of farms decreased by 19% while average herd size increased by 147% (Statistics New Zealand 2012). ‘Mega-dairies’ housing 700 or more animals are only now appearing in the EU (FAO 2018a), while in the US West 90% of dairy farms had more than 500 cows in 2012 (MacDonald et al. 2016).

Transitions in dairy systems are influenced by structural factors such as government subsidies and regulations as well as by social, cultural, economic, and environmental processes. In 2010, cost of production per litre of milk was more than three times higher for farms with fewer than 50 cows than for farms with more than 2000 cows, a substantial gap that meant negative net returns for farms with fewer than 1000 cows (USDA 2010). The emphasis on economies of scale is further upheld by strong state support for industrial operations (Freidberg 2009). Dairy systems in the EU, NA, and ANZ operate through established ‘industrial-commercial complexes’ (Jay 2006): assemblages of infrastructure, technology, and education that facilitate commercial/industrial production and processing and that influence farm management decisions along productivist pathways.

Global markets and international trade agreements have further entrenched intensification as the dominant pathway of dairy system change. Recent pressures to compete in a global marketplace for commodity milk due to trade liberalization policies and skyrocketing dairy production in Brazil, India, and China have spurred deregulation and ‘hyper-productivism’ as dairy producers struggle to achieve efficiency (Dibden et al. 2009). Deregulation occurred decades ago in NZ and the USA and more recently in the EU with the removal of milk production quotas in 2015, a move that is expected to make it difficult for smaller farms to compete (Salou et al. 2017). Dairy supply chains are controlled by a few large processors Dairy Farmers of America in the US and Groupe Lactalis in France) who collect and pasteurise the milk and by supermarket chains (such as Walmart), which control a large majority of milk sales (Jay and Morad 2007). Indeed, by 2017 the 20 largest (by volume) milk processors controlled more than 25% of market share in worldwide milk production (IFCN 2018). Dairy farmers thus have little or no control over milk prices and, in the absence of adequate subsidies, have needed to intensify production or shift to higher value products such as organic, direct sales, or making cheese or yogurt.

### Nonlinear dynamics of dairy system change

Approaching intensified agriculture as a binary production strategy can unproductively smooth the unevenness—between farms, regions, and various stakeholders in a sector—that emerges amid rapid agrarian change (Wilson 2008). Dairy intensification should be considered a complex process that unfolds in places over time. There is a need for studies exploring the socioecological struggles that emerge amid this complex and uneven change. For example, research could investigate how transitions towards intensive production as well as alternative production modes (such as organic) can eschew agro-industrial modes of production or, alternately, how they may recreate aspects of conventional production systems (cf. Guthman 2004).

Given the complex and political nature of dairy system transitions, research should consider how structural aspects (such as farm size, supply chains, and policies) can intersect with on-farm social and environmental contexts (Wilson and Burton 2015). While recent work has investigated the uneven human–environment outcomes surrounding transitions in meat production systems (Emel and Neo 2015; Neo and Emel 2017), similar research on dairy is notably lacking. In the next section, we aim to build the foundation for such work by reviewing the effects of dairy system intensification along four key dimensions highlighted in the literature.

## Effects of dairy intensification

Research on dairy intensification has primarily considered its effects in four areas: (1) the environment; (2) animal welfare; (3) social and economic well-being; and (4) human health. Based on a review of the literature, we summarize and provide examples to illustrate the general findings and principal disciplinary associations of concerns arising in each of these areas.

### The environment

The environmental impacts of dairy production can vary substantially depending on farm management practices (Poore and Nemecek 2018). The increased reliance of intensive dairy systems on inputs can exacerbate some negative environmental effects, both directly and indirectly, and alleviate others (Eshel et al. 2014). Commonly studied environmental impacts of dairy systems include emission of greenhouse gases (GHGs); soil and water pollution; biodiversity loss and wildlife health; nutrient cycles (primarily nitrogen and phosphorus); and land use change.

Dairy production gives rise to the emission of three GHGs: carbon dioxide (CO_2_, via energy use and land use change), nitrous oxide (N_2_O, from feed production and excreta), and methane (CH_4_, enteric and from manure). The process of intensification can increase some emissions while reducing others. As such, results are mixed as to how intensification broadly affects GHG emissions. Some studies suggest that more intensive and consolidated dairy production can decrease overall GHG emissions (Clark and Tilman 2017). On the other hand, some argue that compared to grazing-based systems (which may store and sequester carbon in grasslands in some contexts) more intensive dairy operations generate higher indirect carbon emissions because of their greater reliance on imported feed (O’Brien et al. 2014; Battini et al. 2016). Others, however, find that even when feed production is considered, intensive systems use less land overall, and as such are implicated in less land-use change related CO_2_ release, leading to lower overall emissions per unit of output (Gerssen-Gondelach et al. 2017).

Intensive dairy production leads to soil and water pollution. Synthetic fertilisers (especially nitrogen and phosphorus) are used to produce feed (generally maize, soy, and barley) and supplements (Cederberg and Mattsson, 2000; Foote et al. 2015). The increased use of fertilizers (organic and inorganic), use of water, and manure disposal issues of large farms can lead to high concentrations of nitrogen, phosphorous, and animal waste. If these are not adequately managed, they can pollute soil, river systems, and shallow aquifers, damaging ecosystems and decreasing the quality of freshwater (Scarsbrook and Melland 2015). While productivity increases mean that less land is needed per unit of milk produced, the pollution from that land increases (Gerssen-Gondelach et al. 2017).

Intensive dairy production impacts biodiversity and ecosystem health directly (through land use changes on-farm) and indirectly (through feed production processes and associated land conversion off-farm). Their reliance on monocultural pasture and high use of fertilizers mean that intensive dairy systems present risks to biodiversity and ecosystem stability in traditionally biodiverse grasslands (De Lucia et al. 2017). Grassland homogeneity can decrease species diversity and richness. For instance, studies in France show that avian populations are less diverse nearer to more intensified livestock production (Dross et al. 2017). Research in Italy similarly indicates decreased butterfly species richness with transitions to more intensive dairy systems (Jerrentrup et al. 2016). On the other hand, grazing-based dairy systems have an opportunity cost in that more land devoted to pasture means less overall land that could be set aside for nature conservation (Cederberg and Mattsson 2000).

### Animal welfare

Discussions about animal welfare generally begin with an often-unarticulated ethical assumption that it is morally acceptable for humans to use animals so long as they ensure that animals are free of physical and mental stress and able to experience positive feelings (Buller and Morris 2003). By contrast, animal rights movements consider any use of animals to be morally objectionable and argue for the development of dairy and meat alternatives (Garner 2016). Understandings of animal welfare tend to include consideration of three aspects: (1) that welfare comprises animals’ essential health and functioning (i.e. absence of disease and injury); (2) the need to consider animals’ ‘affective states’ (such as pain, distress, and pleasure) and how positives and negatives add up to a quantitative indicator of well-being; and (3) animals’ freedom to pursue ‘natural’ behaviours (e.g. grazing in open air), including their ability to exercise control in a given situation to remove themselves from ‘poor’ situations and place themselves in more positive mental and physical states (Fraser 2008; Ohl and Van der Staay 2012). In animal welfare regulations, definitions often combine elements of these positions, encouraging quantification of states of well-being experienced by animals in a mental as well as physical sense (Dawkins 2008). In practice, however, policymakers tend to emphasise absence of disease and injury, which is arguably simpler to measure.

It is possible to have high and low animal welfare in all production systems, including in those defined as extensive, organic or intensive. However, in very intensive confined feeding operations there are arguably limits to how ‘good’ welfare can be (Webster 1994). Rapid intensification in the dairy sector can have considerable impacts on animals’ physical and mental well-being, particularly in high-income countries, where measures to improve productivity deliver only moderate gains, often at the expense of animal welfare (Haskell et al. 2006; LeBlanc et al. 2006). In intensive operations, cows often lack freedom to perform natural behaviours of grazing, reproducing, and socializing in pasture but instead live in housing regimes that constrain movement and that require animals to stand on concrete floors for extended periods of time (von Keyserlingk et al. 2009). Intensive dairy systems have been shown to have a higher prevalence of lameness and other disease (Koeck et al. 2014). Breeding cows for higher productivity also exacerbates physical and emotional stress on animals, thereby decreasing their welfare (Oltenacu and Broom 2010). Moreover, the lower levels of interaction between cows and stockmen that are common on intensive farms can increase the risk that animal welfare issues go unnoticed (Burton et al. 2012), although on the other hand larger farms may have greater access to specialised veterinary care. Management strategies that aim to optimize milk productivity can negatively impact animals’ life cycles. For example, in intensive operations, cows are artificially inseminated again shortly after they have given birth to a calf, and then slaughtered after only a few pregnancy-lactation periods (von Keyserlingk and Weary 2017).

### Livelihoods and well-being

The intensification of dairy farming has complex and uneven effects on various social, cultural, and economic dimensions of human well-being. Findings vary depending on the disciplinary framing and the scale of analysis. Economists tend to consider the monetary costs and benefits of various milk production regimes, often conducting national-level analyses of the structural factors influencing transitions and impacts in dairy systems (e.g. Zimmermann and Heckelei 2012). At the national level, there can be demonstrable economic benefits of increasing dairy farm productivity. For instance, in New Zealand dairy exports increased by 460% between 1990 and 2012 due to productivity increases from intensification, leading to gains in Gross Domestic Product (GDP) (Statistics New Zealand 2012). In identifying macro-level economic benefits of intensive systems, this work can generate support for policies that incentivize further dairy intensification (Mcgregor and Houston 2017).

On the other hand, sociologists, anthropologists, and geographers tend to consider broader socio-cultural and political economic issues surrounding intensification at more local levels, speaking to fundamental ideological debates concerning the role of agriculture in an increasingly urbanised world (Galt 2013). This work has investigated issues such as power asymmetries that are inherent in an industrial agricultural model, the loss of small scale farms due to price competition, the replacement of traditional skills and ways of life with a corporate mindset, and how trading regimes can undermine small scale production (Davidson 2002; Diamond 2013; Krieg 2014). Studies at local and regional levels demonstrate that the drive to increase output amid falling milk prices can push small and medium-size farms out of business if they are unable to restructure their enterprises (Davidson and Schwarzweller 1995; Krieg 2014). This can have cascading effects in that family farming becomes less viable, rural employment opportunities decrease, and cultural landscapes may disappear (Davidson 2002).

Such work generally finds that transitions to intensive dairy production systems can have uneven impacts in rural areas, leading to marginalization of some households across and within production regions. For example, Davidson and Schwarzweller (1995) demonstrate the nuances of marginalization and the vulnerable position of dairy producers in the Midwest US to rapid shifts in markets or policies. Another common finding of research on dairy system intensification is that the cost savings that are achieved in more intensive operations can in part be attributed to lower human labour input, which generally results in losses of employment for family and non-family dairy workers (Davidson 2002). As a case study from Norway demonstrates, these losses of rural employment opportunities have the greatest negative impacts on family members and communities in areas distant from urban centres where the bulk of milk is produced on small farms and where there are fewer non-farm livelihood options (Flaten 2002). Some have considered gendered impacts of dairy intensification on family farms, illustrating how intensification can validate men’s roles as ‘farmer-managers’ while diminishing the value of women’s contributions to farm household labour burdens (Alston et al. 2017). Other recent work explores how marginalization occurs due to a confluence of biophysical and social factors. For example, Ricard (2015) demonstrates that in the absence of EU milk production quotas, greater competition and production gaps between highland and lowland areas have made it challenging for producers in mountainous areas to remain in business.

### Human health

The links between dairy intensification and human health are complicated and contested. Health is more than the absence of disease and has long been defined as the ability to achieve states of physical, mental, and social well-being (WHO 1946). In shaping what people eat, the cultural, economic, political, and environmental dynamics of food systems have a major influence on population health: around 2 billion people have nutritional deficiencies and another 2 billion are overweight (WHO 2018). At the same time, the links between health and diet have become increasingly prevalent in scientific research and public policies (Springmann et al. 2016). Intensive dairy systems can present direct risks to human health through acute and chronic soil, air, and water pollution as well as by increasing exposure to zoonotic diseases, pathogens, and exacerbating risk of anti-microbial resistance (Wing and Wolf 2000). Dairy intensification can also have indirect impacts on human health, which from the level of the individual to populations are hotly contested. On the one hand, by reducing the cost of dairy products (which are important sources of protein and micronutrients), intensive dairy production may contribute to the nutritional adequacy of diets, a point long heralded in the US milk industry (Dupuis 2002) and in international development policies (FAO 2018a). On the other hand, the overabundance of low-cost dairy products may reduce dietary diversity and lead to increased cardiovascular disease (Alexander et al. 2016). However, other recent studies indicate weak or no relationship between milk, butter, and cheese consumption and cardiovascular disease, suggesting that dairy products contain amino acids, vitamins, minerals, and fats that may in fact provide a net health benefit (Dehghan et al. 2018).

While the links between dairy and health remain inconclusive, food systems continue to be marked by inequities in ability to access nutritious, healthy food (Allen 2008). Combined with high socioeconomic inequality and pre-existing health disparities, the lack of access to healthy food has led to higher rates of obesity, heart disease, cancer, and diabetes (Alkon and Mares 2012). Neoliberal agricultural policies have further concentrated power with agro-industry such that the current food regime is rife with health injustice: food insecurity, malnutrition, and diet-related health issues arise disproportionately among black, latino, and low-income communities (Guthman 2011). Given the complexities and the political nature of links between dairy intensification and human health, it is essential to consider the social, economic, and cultural contexts of dairy consumption and production and how they generate uneven health outcomes.

### Synergies and trade-offs across dimensions in dairy production-consumption

**Fig. 1:**
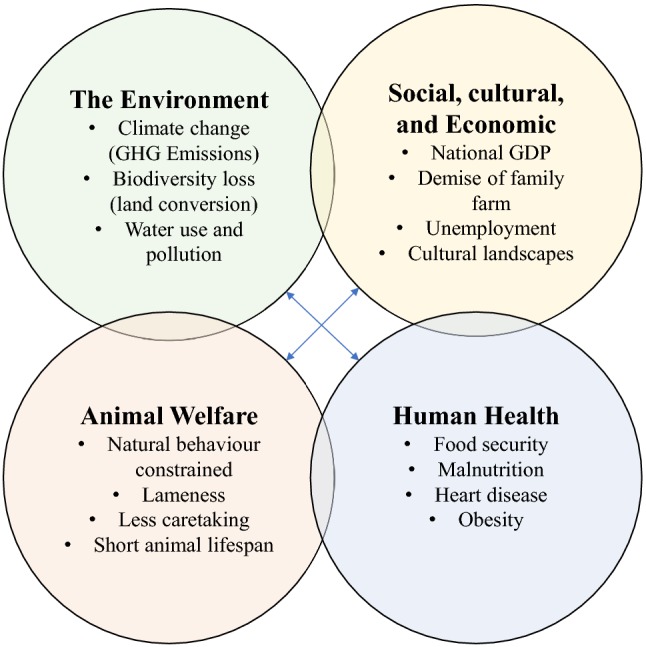
The impacts of dairy intensification along four dimensions

Far less research has considered how these multiple dimensions (categorized in Fig. 1) of dairy intensification interact. This presents a critical limitation in understanding the synergies and trade-offs of current and future dairy systems. However, some recent work has considered how trade-offs and synergies can emerge. As an example of negative synergies between economic and environmental effects, research in Ireland indicates that intensified dairy operations using imported concentrate feed increase the carbon footprint of milk while decreasing farm profit margins due to only incremental increases in per-cow productivity (O’Brien et al. 2015). As an example of trade-offs between economic benefits and health outcomes, research indicates that the predominant economic procedure of valuing agriculture in terms of GDP does not account for its environmental or health impacts, distorting market prices for food in ways that can lead to overproduction and overconsumption (Pretty et al. 2000; Tilman and Clark 2014). Other work suggests the potential for positive synergies between health and the environment, where curbing consumption of meat and dairy in the EU would likely improve environmental outcomes by reducing resources devoted to their production (Westhoek et al. 2014; Springmann et al. 2016).

Research has also considered how environmental and political economic contexts (including policy responses to environmental change) intersect to shape outcomes in dairy systems. For example, drought and concerns for water quality and river health have led to policies in Australia that restrict water usage on irrigated dairy farms, resulting in altered livelihood strategies and gender relations that—together with the impacts of climate change—have reduced farm productivity (Alston et al. 2017). As an example of trade-offs between climate change mitigation policies and animal welfare, a recent study suggests that intensification is often promoted as a route to improving GHG efficiency of dairy; however, this can be at a cost to animal welfare (Shields and Orme-Evans 2015). Importantly, the benefits and trade-offs of food system adjustments are context specific, emerging at both regional and global levels (Springmann et al. 2016). This suggests the need for interdisciplinary research and policy on dairy systems that can reconcile complex multi-scalar (that is, local to global) processes.

## Framing dairy transitions

Recognition of these negative impacts of intensification has led to efforts to envision and enact alternative dairy futures. These efforts can be categorized into three broad framings that shape research and ongoing policy and advocacy agendas on dairy production and consumption: (1) sustainable intensification, (2) multifunctional agriculture (including alternative food networks), and (3) agroecology. These framings are compared in Table 1. We consider the relative merits of each of these alternatives to conventional dairy systems. It is important to recognize that these framings are in flux. They are defined by various groups in different ways and are contested among those who employ them. Moreover, while these framings have been considered in some detail in work on agriculture at large, they have not featured substantially in research and policy on dairy systems. We begin to address this gap by considering how these framings broadly orientate dairy research and policy. For each framing, we (1) provide a general description, noting briefly if and how it has been employed in research and policy on dairy; (2) discuss how well the approach may be positioned to assess the multidimensional and interlinked environmental, animal welfare, socioeconomic, and human health related issues of dairy systems (as discussed above); and (3) consider how the framing envisions dairy futures and the degree of systemic change—from subtle tweaks to more radical transformation—contingent upon those futures. Having considered the relative merits and drawbacks of the three alternative framings, we then develop suggestions for future research and policy.

**Table 1:** Comparing the relative merits of the three research/policy framings for alternative dairy systems

	Sustainable intensification	Multifunctionality	Agroecology
*Premise*	Need to increase food production while reducing resource use; characterized by supply-side tweaks to enable lower resource use that conserves biodiversity and reduces GHG emissions	Rural landscapes more than food production: also have cultural and environmental value; characterized by changes to production-consumption networks that account for these values	Self-sufficient production-consumption systems (minimizing external inputs) that optimize local knowledge of biophysical elements to enhance food sovereignty and justice
*What does the system look like?*	Very large farms; investment in new technologies to minimize resource use (land, water, nutrients) and GHG emissions and to better manage nutrients to prevent water pollution; common mechanisms include genetic and other modification of animals and feed as well as grazing management	Organic or ‘alternative food networks’ such as community supported agriculture, which shorten supply chains and add value, often through substituting organic inputs; schemes of payments for environmental services such as planting trees; productivity often lower than conventional	Diversified farming that includes crops and livestock; ecologically grounded techniques such as conservation tillage, green manure, biological pest control, agroforestry, rainwater harvesting; farmer empowerment through learning and adaptive decision making
*Does it address multiple human*–*environment dimensions of dairy systems*?	Economic and some environmental issues (e.g. GHG emissions); little about health and social issues, or animal welfare	Social and economic; some environmental aspects (e.g. ecosystem services); little about animal welfare or health	Environmental, political, and social; less attention to health and animal welfare
*How feasible is it to implement as policy*?	High feasibility due to technological nature of changes and continued productivist mindset	Moderate feasibility due to simple adjustments to production systems; but inadequately defined	Low feasibility due to challenges of scaling up and mainstreaming in policies that are structured around industrial agriculture
*What transformation does it suggest or require*?	Low level of food system transformation, mostly relevant for large farms; fixes mainly technical	Moderate transformation needed; Mostly relevant for small and medium farms	High level of transformation; mostly relevant for small, mixed crop-livestock systems
*What change to consumption does it suggest or require*?	Low change to amount of consumption; designed to meet business as usual predicted increased food demands of growing consumption	Moderate change: changing type of consumption (e.g. more organic, local, artisanal dairy), but little about amount of dairy consumed	Moderate change: need to reduce consumption for this model to work

### Sustainable intensification

One proposed strategy for mitigating the negative environmental impacts of conventionally intensive agriculture is sustainable intensification (SI). SI emerged as a response to the acknowledged need to increase food production to meet growing demand and the recognition that agricultural production can have negative environmental impacts (Pretty et al. 2011). To increase food production without impinging on resources and the environment, SI emphasizes the importance of increasing productivity per unit land (Godfray et al. 2010). SI has been employed worldwide. It features in agricultural policies of numerous countries and as an organizing concept of international organizations, yet it is defined in different ways by different groups (Godfray 2015). As applied in diversified farming systems of lower-income countries in Africa, for example, some maintain that SI can increase agricultural productivity through a combination of external technical knowledge and local agro-ecological knowledge (Pretty et al. 2011). In this sense, SI merges a productivist mindset with appreciation of biophysical and social variations in farming landscapes and emphasises the valuable role of small farm families and their local knowledge in achieving higher agricultural yields in socially and environmentally sustainable ways (Vanlauwe et al. 2014).

However, as applied in the global North, SI tends to be interpreted quite differently. While SI is a nascent concept in the dairy sector, it is often framed as a response to issues of inefficient production, where minimizing negative environmental impacts can enhance productivity (Soteriades et al. 2016). In turn, sustainability has tended to be conceptualized as something that can be achieved by enhancing the efficiency of existing operations through use of scientific and technological advances, with little attention paid to local agro-ecological knowledge and social-environmental variation. SI strategies in the dairy sector tend to target environmental issues that are also seen as economic inefficiencies (e.g. reducing GHG emissions by breeding cows to emit less methane or planting grasses that require less nitrogen fertilizer [Foresight 2011]). In practice, SI’s arguably narrow framing of increasing production without increasing resource use or environmental impact makes it somewhat poorly positioned to address the multidimensional issues associated with dairy systems, particularly animal welfare, human health, and social elements. Although in theory SI diverges from conventional input-based intensification in terms of the need to reduce consumption (Godfray 2015), in practice it is limited by its supply side emphasis on increasing agricultural efficiency through advances in technology and management (Tilman et al. 2011).

There is a recognized need to consider how to derive more holistic benefits from SI that go beyond increasing aggregate crop yields (Garnett et al. 2013). Some have expressed concern that SI has become merely another iteration of the ‘sustainability’ buzzword, more akin to greenwashing than to fundamental transformation of agricultural systems (Loos et al. 2014). Questions remain about how to balance trade-offs between sustainability and productivity and what is meant by environmental efficiency (Godfray 2015). With its techno-scientific approach and focus on environmental and economic issues, there may be limits to how far SI can be expanded to encompass multidimensional concerns such as livelihoods, animal welfare, and health. Perhaps the worst-case scenario would be that employing SI as a research and policy framing bolsters a narrow conceptualization of dairy transformation as a drive towards greater economic-efficiency. That could divert attention from the complex social-ecological processes that shape the multidimensional impacts of dairy production. It could also risk enabling the highest-impact dairy producers to continue relatively unchanged, while affording lower-impact producers (i.e. less intensive operations) little opportunity to further reduce impacts. In these ways, SI could further entrench conventional production practices and contribute to pushing smaller farms out of business (Vanloqueren and Baret 2009). Nevertheless, components of sustainable intensification may prove valuable in addressing some of the negative impacts of dairy intensification, particularly if applied together with insights from other framings.

### Multifunctional agriculture

A second framing is multifunctional agriculture, a *non*-*productivist* paradigm wherein rural landscapes are valued for diverse services beyond agricultural production (Wilson and Burton 2015). As a policy platform, multifunctionality often reintegrates agriculture with rural development initiatives (e.g. reducing poverty and enhancing livelihoods) and merges these with agendas of environmental protection (Wilson 2007), thereby shifting from a sectoral focus on agriculture to a more holistic focus on regions (Marsden and Sonnino 2008). In theory, this regional focus enables policies to target human–environment components of multifunctional agricultural landscapes, such as helping rural landowners diversify their livelihoods, contribute to non-agricultural ecosystem services (e.g. biodiversity or climate change mitigation), and adopt mechanisms to generate further value from production such as by converting to organic production or developing closer and more direct linkages with consumers in order to capture a greater share of product value (Rache and Argent 2015). Multifunctional landscapes also emphasize the upkeep of social and cultural services, such as family farming and pastoral aesthetics of rural landscapes, which have value for rural communities and for agritourism (Freidberg 2009).

Multifunctional agriculture has often emerged organically amid intensification and concentration of dairy production. Smaller producers have remained in business by diversifying livelihoods (e.g. working in off-farm jobs) or by making changes to add value to existing production models (e.g. converting to organic or providing ecosystem services) (Davidson and Schwarzweller 1995; Krieg 2014). Although there is growing attention to alternative food networks and conversion to organic dairy production, little work has addressed multifunctionality in dairy systems specifically. In theory, the multifunctional agriculture framing considers multiple social and environmental dimensions. Likewise, in its aim to shift towards a broader conception of rural landscapes than merely places where food and fibre are produced, multifunctional agriculture appears to call for more fundamental transformations to production systems than does SI.

However, in practice multifunctional agriculture often takes the form of environmental and amenity schemes (such as CAP greening measures that pay farmers for planting hedgerows). Programs to enhance multifunctionality of agriculture have drawn criticism for hewing to predominantly economistic measures of success (such as quantifying biodiversity as an ecosystem service), thereby undervaluing more systemic regulating services such as climate and water quality (Garzon 2005). Such policies have arguably failed to offer genuine rural development opportunities for small farmers because they act more as subsidies and can constrict the overall state budget allotted to agriculture (Marsden and Sonnino 2008). Some suggest that multifunctional agriculture risks weakening state regulation by shifting accountability to market mechanisms (McCarthy 2005). Others have demonstrated that in some cases organic milk systems can follow the same path as conventional milk, trending towards domination by agribusiness dairies that employ large confined animal feeding operations (DuPuis 2002). By these accounts, multifunctional agriculture has fallen short of its transformative and multidimensional potential.

### Agroecology

A third framing is agroecology, which came of age in 1930s North America and Europe and is variously defined as a scientific approach, a social movement, and a practice (Wezel and Soldat 2009). As a research approach fusing ecology and agronomy, agroecology has been defined as “the science of applying ecological concepts and principles to the design and management of sustainable food systems,” (Gliessman 2007, p. 369). As a practice, agroecology developed through attention to local ecological knowledge and recognition of socioeconomic factors (Tomich et al. 2011). More broadly still, agroecology has been defined as “the integrative study of the ecology of the entire food system, encompassing ecological, economic and social dimensions” (Francis et al. 2003, p. 100). In considering the social, political, and cultural contexts in which farm management decisions are made, agroecologists often stress the importance of social and political change to enabling food system sustainability and justice (Vandermeer and Perfecto 2017). As such, agroecology is closely aligned with political and social movements such as *La* Via *Campesina*.

Agroecological principles of livestock keeping (e.g. mixed systems that combine livestock with trees and annual crops to enhance overall farm productivity) are widely practiced in low and middle-income countries. Although once common in the global North, similar agroecological practices have been challenging to implement in regions where large-scale intensive dairy production dominates (FAO 2018b). However, there is growing interest in agroecological approaches to dairy production in temperate regions (Bonaudo et al. 2014), where these approaches tend to emphasise the self-sufficiency of farms, particularly as regards animal feed (Lebacq et al. 2015). Other practices include permaculture systems, breeding more multifunctional livestock rather than raising breeds with the highest milk production, or incorporating semi-natural grasslands into mountain agriculture (Dumont et al. 2014).

Agroecology is closely aligned with multifunctional agriculture (Wibbelmann et al. 2013) and similarly aims to create opportunities for smaller producers who have been excluded from benefits of the industrializing dairy sector (FAO 2018b). But agroecology’s practical and multidisciplinary origins set it apart from multifunctionality and SI in terms of its consideration of the multiple dimensions of dairy systems and its emphasis on the need to fundamentally transform food systems as part of a broader agenda of social change (Tomich et al. 2011). As both a scientific discipline and social movement, agroecology emphasises multidisciplinary studies on food systems that span social and natural sciences, close interactions with farmers, and practical engagement with activist groups aiming to catalyse social change (Vandermeer and Perfecto 2017). Of the three framings reviewed here, agroecology is arguably the best positioned to address the uneven power dynamics that underlie trends of dairy intensification (Jay and Morad 2007).

However, there are substantial challenges to mainstreaming agroecology in policy agendas and research programmes (Wibbelmann et al. 2013). The continued domination of productivist paradigms in discussions about livestock production have limited the research funding and agricultural extension activities exploring agroecological approaches (Vanloqueren and Baret 2009; McGregor and Houston 2017). Indeed, the emphasis on optimizing system productivity at the expense of the productivity of individual components (such as milk output) makes agroecology fundamentally at odds with productivist agricultural models that are predicated on specialisation (Dumont et al. 2014). This also has the effect that agroecological approaches to livestock production are challenging to implement at a large scale (Moraine et al. 2014; FAO 2018b). Thus, while agroecology may offer the most radical food system transformation of the three framings, such transformation is predicated on substantial political economic change.

## Towards multidimensional research and dairy system transformations

Not only do these three framings of agricultural transitions elude clear definitions, they are also contested, they overlap conceptually, and in practice they are often found juxtaposed across rural landscapes. For example, in the EU, multiple production systems coexist in a patchwork at local and regional levels; conventional (non-intensive) operations often occur next to organic mixed systems, intensive grassland systems, and intensive maize silage systems (European Commission 2016). Transitions from conventional to multifunctional agriculture occur through heterogenous and nonlinear pathways (Wilson 2008). Likewise, there is substantial overlap between multifunctionality and agroecology, as evidenced by efforts to consider how agroecological principles could help in the design of multifunctional landscapes (Lovell et al. 2010). And agroecological principles can also be applied in SI (Tittonell 2014). Moreover, the decision of which framework to apply is inherently political, as it determines the criteria of research and policies and therefore shapes agrarian and food futures. It is further important to consider how findings and policies might differ depending on the level of analysis that is adopted (e.g. farm, or region, or nation). For example, national and international assessments tend to privilege SI. However, as this review has shown, the processes driving dairy intensification, the multiple dimensions of their impacts, and the policy responses span multiple levels of organization, making cross-scalar research essential.

As discussed in Sect. 3, work on the effects of dairy intensification has tended to isolate social or environmental dimensions. Occasionally, research and policy considers interactions of these dimensions, but generally with little depth, such as by highlighting links between GHG emissions and economic productivity, rather than pursuing more involved research that considers systemic social, political, and ecological processes. Yet, research and policy on environmental issues are often rooted in values, which structure understanding of how food system sustainability is or should be defined (Garnett 2014). For example, in studies rooted in a productivist agriculture ethic, environmental management tends to be circumscribed by a political economy that prioritizes economic competition in a global dairy market (Jay 2006). There is a need for policy-relevant research that is not beholden to pre-existing overly simple narratives (e.g. productivist or post-productivist), which obscure understanding of the complexities of dairy system change and its multidimensional impacts.

Given the complex nature of dairy systems and the interrelated social-ecological processes, policies working within paradigms of sustainable intensification and multifunctionality have limited prospects for success. Policies rooted in these frameworks tend to target single dimensions of dairy system impacts, overlooking complex overlaps among the multiple dimensions. For example, sustainable intensification addresses environmental aspects while paying less attention to animal welfare, human health, and rural development (Garnett 2014). Moreover, despite ever-increasing specialization in the dairy industry, agricultural policies are often not tailored to the specificities of dairying. We suggest that there is a need for governance mechanisms that can address the unique issues of dairy systems and that are also flexible enough to account for these systems’ spatial and temporal variations. The need for proactive policies regulating dairy systems is evidenced by the variations in the environmental impacts of various production models (Poore and Nemecek 2018).

This review on the causes and impacts of dairy intensification highlights a clear and urgent need for systemic transformations. The rapid intensification of dairy production has exacerbated CO_2_ emissions, animal welfare abuses, inequities in human health, and the eroding of rural livelihoods and cultural landscapes. Yet transformation is itself a political process. Policies aiming to influence transformative adaptation within dairy systems should be informed by analysis of power differentials in effort to gauge the equity of agricultural policies (Alston et al. 2017). Recognition that dairy production affects the environment, health, and animal and human welfare in complex and nonlinear ways must come alongside investigation of the politics surrounding how these dimensions are defined and assessed. In other words, what definitions, metrics, units, and scales are employed? Comprehensive studies that consider how these outcomes emerge at the interface of broader structural change (e.g. shifting trade policies) and heterogeneous ‘local’ social-environmental contexts are especially needed (Galt 2013).

Specifically, research is needed that considers dairy landscapes within broader processes of socioecological change. This work should document the uneven social and environmental outcomes that emerge during dairy system transitions, whether these are transitions to more intensive production or towards more multifunctional or agroecological systems. This focus on social inequalities will help inform transformations to more just food futures. The transitions that have occurred to this point within dairy systems have been scripted by powerful entities, including dairy processors and retailers. This political economy has restricted alternative strategies like agroecology and community-supported agriculture and has rarefied approaches like SI and organic agriculture, by making these palatable to conventional dairy operations. This has meant incremental, site-specific changes rather than systemic changes to the food system that can also address entrenched inequalities. In reviewing the complexities and intersections of multiple elements of dairy system change, this paper may help reimagine more just and sustainable pathways of change in of dairy systems.
